# Posterior Reversible Encephalopathy Syndrome With Spinal Cord Lesions Associated With Malignant Hypertension and Post-renal Transplantation Rejection in a Patient With IgA Nephropathy: A Case Report

**DOI:** 10.7759/cureus.82564

**Published:** 2025-04-19

**Authors:** Jun Soma, Jun Sawada, Tomohiro Uemura, Shiori T Kikuchi, Naoki Nakagawa

**Affiliations:** 1 Division of Respiratory Medicine and Neurology, Department of Internal Medicine, Asahikawa Medical University, Asahikawa, JPN; 2 Division of Cardiology, Nephrology, Pulmonology, and Neurology, Department of Internal Medicine, Asahikawa Medical University, Asahikawa, JPN

**Keywords:** chronic antibody-associated rejection, iga nephropathy, malignant hypertension, posterior reversible encephalopathy syndrome, spinal cord

## Abstract

A 40-year-old Japanese male patient was admitted to the emergency room because he had been experiencing visual disturbances in both eyes for a month, and his fatigue had worsened over the past week. He was diagnosed with IgA nephropathy 24 years ago and underwent a living donor kidney transplant 19 years ago. The patient was administered tacrolimus and mycophenolate mofetil (MMF) since the renal transplantation. Upon admission, his blood pressure (BP) rose to 250/150 mmHg. Neurological examination revealed bilateral light perception, left homonymous hemianopsia, bilateral patellar hyperreflexia, and decreased sensation of vibration below the lumbar region. Brain magnetic resonance imaging (MRI) showed hyperintense areas on both fluid-attenuated inversion recovery (FLAIR) and apparent diffusion coefficient (ADC) maps in the right parieto-occipital lobe, left basal ganglia, and white matter around the lateral ventricles, brainstem, and bilateral cerebellar hemispheres. Some lesions showed hyperintense areas on the diffusion-weighted image (DWI). MRI of the spinal cord on T2-weighted images showed hyperintense areas in the center of the entire spinal cord. The patient was diagnosed with posterior reversible encephalopathy syndrome with spinal cord involvement (PRES-SCI) and was treated with antihypertensive therapy, immunosuppressive drugs, and dialysis. The patient’s clinical symptoms and imaging findings gradually improved. A follow-up MRI of the brain and spinal cord on day 43 after onset showed almost complete disappearance of the lesions. This is the first reported case of PRES-SCI involving the entire spinal cord in a patient with renal disease. In this case, malignant hypertension, post-transplantation rejection, and immunosuppressive medications may have been involved in the onset of PRES-SCI. Prompt diagnosis and treatment may lead to favorable outcomes.

## Introduction

Posterior reversible encephalopathy syndrome (PRES) was first reported in 1996 by Hinchey [1]. PRES is a reversible cerebral white matter encephalopathy characterized by vasogenic brain edema [1]. Patients with PRES can present with acute neurological symptoms such as headache, seizures, consciousness disturbance, and visual disturbance [2]. PRES occurs in patients with renal failure, blood pressure (BP) changes, immunosuppressive medications after organ transplantation, chemotherapy, autoimmune diseases, eclampsia, pre-eclampsia, and sepsis [3-5]. PRES is associated with kidney dysfunction in 55% of patients [6]. Among these cases, those with spinal cord involvement are considered to have PRES with spinal cord involvement (PRES-SCI) [7]. We report a patient with IgA nephropathy and PRES-SCI who was treated with antihypertensive therapy and hemodialysis and left with no neurological sequelae. To the best of our knowledge, this is the first PRES case involving the entire spinal cord in a patient with renal disease.

## Case presentation

The patient, a 40-year-old Japanese male, was admitted to the emergency room due to visual disturbances in both eyes for a month, and his fatigue had worsened over the past week. He was diagnosed with IgA nephropathy 24 years ago and underwent a living donor kidney transplant 19 years ago. His renal dysfunction had progressed gradually over the past three years. He had been diagnosed with chronic antibody-associated rejection nine months prior, and the dose of immunosuppressive drugs had increased. He received tacrolimus (2.4 mg/day) and mycophenolate mofetil (MMF) (750 mg/day). He had poorly controlled hypertension for several months. His BP was usually between 160/90 and 170/100 mmHg but increased to 250/150 mmHg on admission. He was conscious and alert. Neurological examination revealed bilateral light perception, left homonymous hemianopsia, bilateral patellar hyperreflexia, and decreased sensation of vibration below the lumbar region. Urinalysis revealed hematuria and proteinuria, and blood tests revealed anemia (Hb 6.6 mg/dL), crushed erythrocytes, thrombocytopenia, and renal dysfunction; creatinine level was 6.69 mg/dL (Table 1).

**Table 1:** Laboratory data at the time of admission. PTINR: prothrombin time-international normalization ratio; APTT: activated partial thromboplastin time; FDP: fibrinogen/fibrin degradation products; MMF: mycophenolate mofetil; sIL-2R: soluble interleukin 2 receptor; dsDNA: double-stranded DNA; MPO-ANCA: myeloperoxidase-antineutrophil cytoplasmic antibody; PR3-ANCA: proteinase 3-antineutrophil cytoplasmic antibody; ACE: angiotensin converting enzyme; ADAMTS13: disintegrin and metalloproteinase with a thrombospondin type 1 motif, member 13; AQP4: aquaporin 4; MBP: myelin basic protein; OCB: oligoclonal band; MOG: myelin oligodendrocyte glycoprotein; CBA: cell-based assay; RBCs: red blood cells.

Blood chemistry		Peripheral blood	
Total protein (6.6-8.1 g/dL)	5.0	White blood cells (3.30-8.60/µL)	6,690
Albumin (4.1-5.1 g/dL)	2.7	RBCs (4.35-5.55×10⁴/µL)	229
Total bilirubin (0.4-1.5 mg/dL)	0.6	Hemoglobin (13.7-16.8 g/dL)	6.6
Aspartate aminotransferase (13-30 U/L)	25	Hematocrit (40.7-50.1%)	18.6
Alanine Aminotransferase (10-42 U/L)	10	Mean cell volume (83.6-98.2 fL)	81.5
Amylase (44-132 U/L)	156	Crushed erythrocyte	1+
Alkaline phosphatase (38-113 U/L)	64	Platelet (15.8-34.8×10⁴/µL)	7.1
γ-Glutamyltransferase (13-64 U/L)	17		
Creatine kinase (59-248 U/L)	298	Coagulation study	
Lactate dehydrogenase (124-222 U/L)	999	PTINR (0.80-1.20)	1.05
Urea nitrogen (3.7-7.0 mg/dL)	114.5	APTT (27.0-39.9 second)	32.8
Creatinine (0.65-1.07 mg/dL)	6.69	Fibrinogen (160-350 mg/dL)	317
Sodium (138-145 mEq/L)	130	FDP (0.0-9.9 µg/mL)	31.8
Potassium (3.6-4.8 mEq/L)	5.6	D-dimer (0.00-0.50 µg/mL)	32.3
Chlorine (101-108 mEq/L)	104		
Calcium (8.8-10.1 mg/dL)	7.4	Drug blood levels	
Phosphorus (2.7-4.6 mg/dL)	7.4	Tacrolimus (ng/mL)	3.16
Fasting glucose (73-109 mg/dL)	105	MMF (μg/mL)	3.8
C-reactive protein (<0.14 mg/dL)	<0.10		
sIL-2R (122-496 U/mL)	952	Urinalysis	
Immunoglobulin G (861.0-1747.0 mg/dL)	529.2	Protein	3+
Immunoglobulin A (93.0-393.0 mg/dL)	189.4	Occult blood	2+
Immunoglobulin M (33.0-183.0 mg/dL)	121.4		
Antinuclear antibodies (<40)	<40	Cerebrospinal fluid	
Rheumatoid factor (<15.0 IU/mL)	3.0	Pressure (70-180 mmH₂O)	>300
Anti-dsDNA antibody (<10.0 IU/mL)	<0.6	Cells (0-3/µL)	3
Anti-SS-A antibody (<7.0 U/mL)	<0.4	Lymphocytes (/µL)	1
Anti-SS-B antibody (<7.0 U/mL)	<0.5	Neutrophils (/µL)	2
MPO-ANCA (<0.2 IU/mL)	<0.2	Protein (10.0-40.0 mg/dL)	125
PR3-ANCA (<0.6 IU/mL)	<0.6	Immunoglobulin G index	0.58
ACE (7.7-29.4 IU/mL)	10.1	MBP (pg/mL)	468
Lysozyme (5.0-10.2 μg/mL)	51	OCB	negative
Haptoglobin (mg/dL)	16	Glucose (50-75 mg/dL)	65
ADAMTS13 (>10%)	47	sIL-2R (U/mL)	36.0
Anti-AQP4 titers [CBA]	(-)	Interleukin-6 (pg/mL)	144
Anti-MOG titers [CBA]	(-)	Anti-MOG titers [CBA]	(-)

**Figure 1:**
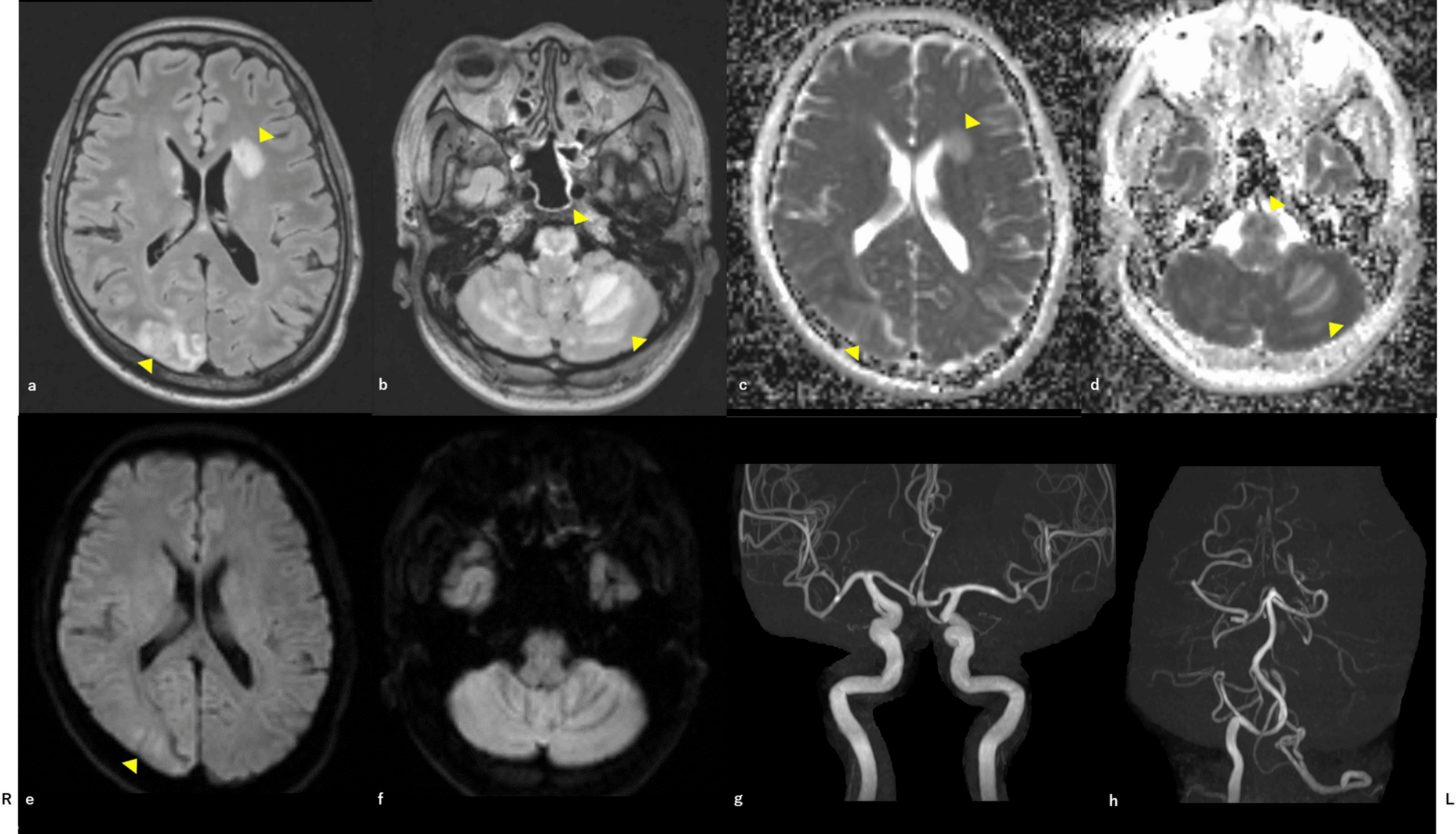
Brain MRI on admission. Axial FLAIR (a, b) and ADC maps (c, d) of the hemisphere. There were hyperintense lesions in the right parieto-occipital lobe, left basal ganglia, and white matter around the lateral ventricles, brainstem, and bilateral cerebellar hemispheres. Axial DWI of the hemisphere (e, f). Some of the lesions are high intensity. Brain magnetic resonance angiography revealed no significant intracranial stenosis of the main trunk artery (g, h). MRI: magnetic resonance imaging; FLAIR: fluid-attenuated inversion recovery; ADC: apparent diffusion coefficient; DWI: diffusion-weighted image.

Disintegrin and metalloproteinase with a thrombospondin type 1 motif member 13 (ADAMTS13) activity, anti-aquaporin 4 (AQP4) antibody, myelin oligodendrocyte glycoprotein (MOG) antibody, collagen-related autoantibodies, and blood levels of tacrolimus and MMF were within normal ranges (Table 1). Cerebrospinal fluid examination (CSF) revealed elevated initial pressure and protein levels, with a normal range of cells. Regarding other CSF examinations, myelin basic protein was elevated, the oligoclonal band was positive, and the anti-MOG antibody was negative (Table 1). Fundus examination revealed Grade IV hypertensive retinopathy. Brain magnetic resonance imaging (MRI) on admission showed hyperintense lesions on fluid-attenuated inversion recovery (FLAIR) and apparent diffusion coefficient (ADC) maps in the right parieto-occipital lobe, left basal ganglia, white matter around the lateral ventricles, brainstem, and bilateral cerebellar hemispheres (Figure 1a-d).

**Figure 2:**
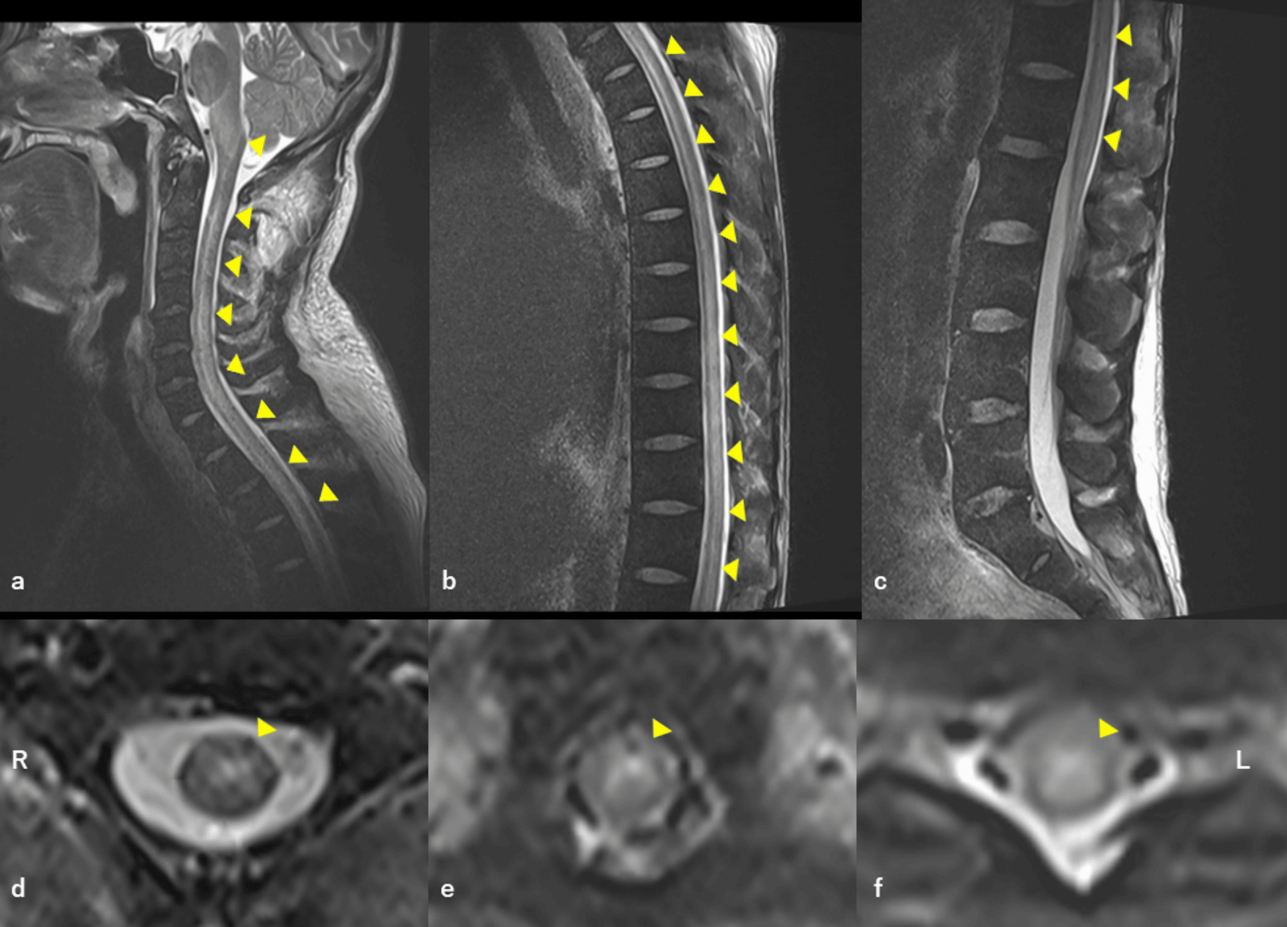
Spinal cord MRI on admission. Sagittal T2-weighted images (a-c). Axial T2-weighted images (d: level of C2 vertebral body, e: level of Th4 vertebral body, f: level of Th10 vertebral body). Long segment (from cervical to lumbar levels) hyperintensity areas in the center of the spinal cord. MRI: magnetic resonance imaging.

Some lesions showed a high intensity on diffusion-weighted imaging (DWI) (Figure 1e, f). Magnetic resonance angiography of the brain revealed no significant intracranial stenosis of the main trunk artery. Spinal cord MRI on T2-weighted images showed long segment (from cervical to lumbar levels) hyperintense areas in the center of the spinal cord (Figure 2)​​​​​​.

**Figure 3:**
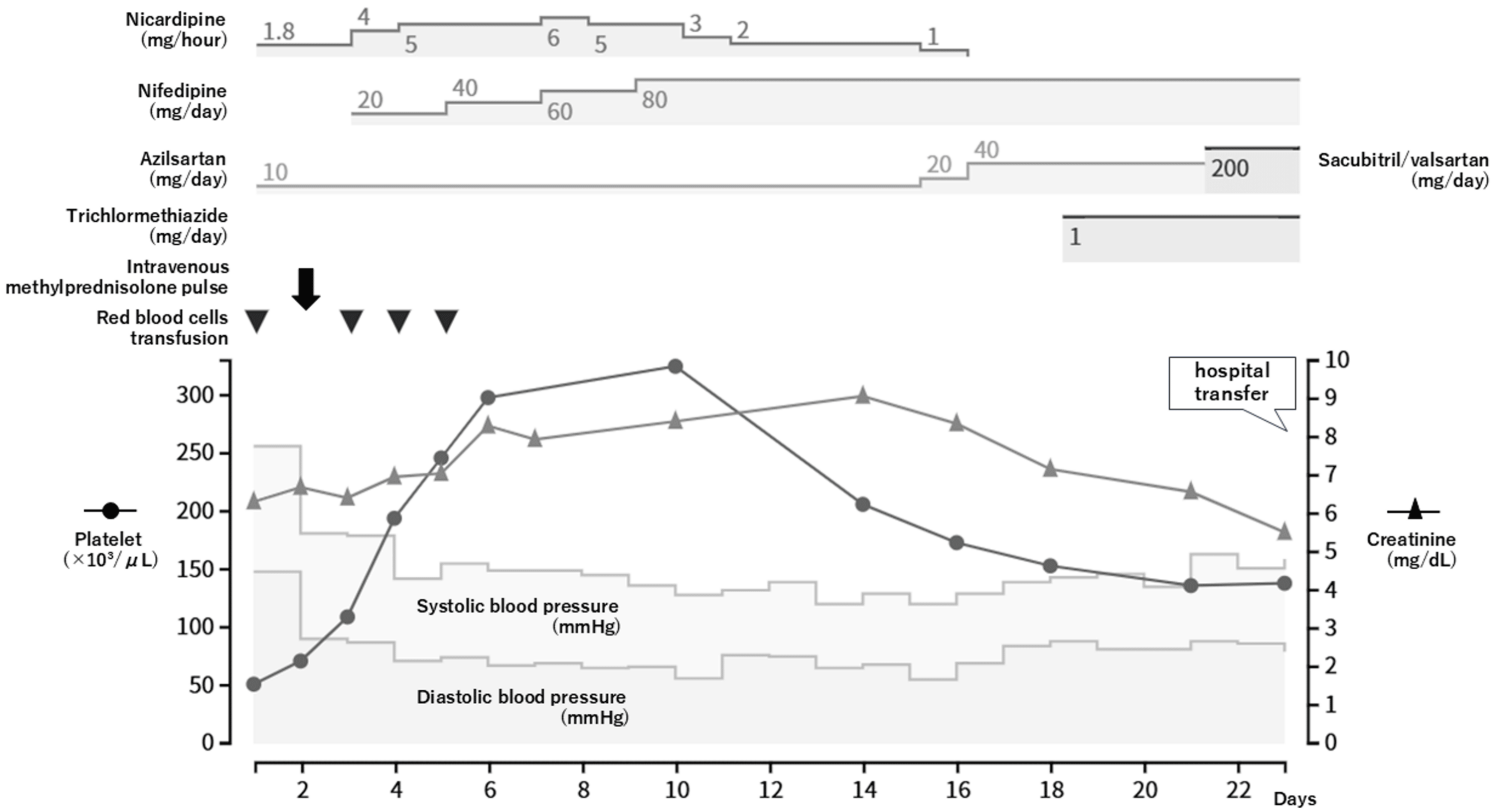
Timeline of blood pressure and platelet, serum creatinine, and antihypertensive therapy.

As PRES was suspected due to significant hypertension and/or immunosuppressive medications, antihypertensive therapy with continuous intravenous nicardipine was initiated, and the doses of tacrolimus and MMF were reduced. Hemolytic anemia and thrombocytopenia were considered secondary thrombotic microangiopathy (TMA), and red blood cell (RBC) transfusions were performed. Hemodialysis was initiated three times per week from the second day of treatment. Considering that neuromyelitis optica spectrum disorder (NMOSD) could not be ruled out, one course of intravenous methylprednisolone pulse (IVMP) was administered with attention to blood pressure elevation. The continuous dose of intravenous nicardipine was gradually reduced and combined with oral nifedipine, azilsartan, sacubitril/valsartan, and thiazide antihypertensive drugs (Figure 3).

**Figure 4:**
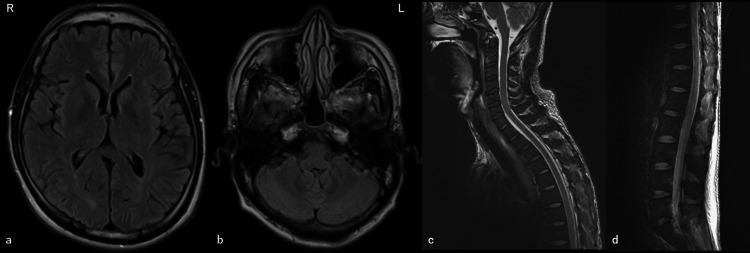
Brain and spinal cord MRI on day 43 after onset. Axial FLAIR of the hemisphere (a, b) and sagittal T2-weighted images of the medulla (c, d). The lesions of the brain and spinal cord had almost disappeared. MRI: magnetic resonance imaging; FLAIR: fluid-attenuated inversion recovery.

His neurological symptoms, including visual disturbances, gradually improved. Similarly, an MRI after treatment revealed a significant reduction in brain and spinal cord lesions. Therefore, the patient was diagnosed with PRES-SCI. The possible triggering factors were acute kidney injury due to malignant hypertension, chronic antibody-related rejection, and the use of immunosuppressive medications. The patient was transferred to another hospital on day 23 and was weaned off hemodialysis on day 27. The brain and spinal cord lesions on MRI almost disappeared on day 43 after onset (Figure 4a-d). Since then, he has not developed PRES.

## Discussion

An association between PRES and chronic kidney disease was reported [8]. Kidney disease is associated with various independent risk factors for PRES, such as hypertension, autoimmune diseases, and immunosuppression. Especially after renal transplantation, PRES is associated with moderate rejection or bacterial infection [9], which may lead to multiple risks such as elevated BP, worsening renal function, and increased use of immunosuppressive drugs. The incidence of PRES varies according to the type of organ transplanted. A retrospective single-center study of 4,222 solid organ transplant recipients showed an overall incidence of PRES of 0.49%, with a 0.35% incidence in kidney transplants [9]. This study showed that kidney transplant recipients with PRES had higher mean BP at presentation than liver transplant recipients but lower grades of vasogenic edema. Although there are many possible causes of PRES in renal transplant recipients, calcineurin inhibitor toxicity is likely the primary cause. However, the toxicity and dose-dependent effects of calcineurin inhibitors remain controversial. Wong et al. reported that most patients with PRES had serum tacrolimus levels within the therapeutic range [10], and there was no correlation between neurotoxicity and tacrolimus trough levels in other studies [11,12].

PRES-SCI frequently occurs in males and is associated with significant hypertension, with symptoms related to spinal cord lesions occurring in half of the cases [7]. The lesion was centrally located in the spinal cord, with no contrast effect or diffusion limitation on MRI. A spinal cord lesion was identified in 5.9% of PRES [13]. As with usual PRES, PRES-SCI resolves within two weeks to six months with treatment of triggers, aggravating factors, and underlying disease [3]. The spinal cord involvement in PRES-SCI is likely to have the same pathophysiology as other classical neuroparenchymal lesions (autoregulatory failure with resultant vasogenic edema); however, other mechanisms may also be operating considering the rarity of PRES-SCI [14]. PRES-SCI is probably underdiagnosed because spinal cord imaging is usually not done in PRES, and symptoms related to spinal cord lesions may not occur frequently. Additionally, PRES-SCI may be misdiagnosed as myelitis. Therefore, PRES-SCI must be differentiated from inflammatory diseases that can cause spinal cord lesions, such as NMOSD, anti-MOG antibody-associated disease, acute disseminated encephalomyelitis, neuro-sarcoidosis, and myelitis due to collagen disease. While treatment for PRES-SCI involves the management of factors causing PRES, such as hypertension, the inflammatory diseases mentioned above are primarily treated with immunotherapy, including steroids and immunosuppressants. Therefore, these diseases should be differentiated as early as possible.

To date, there have been 10 cases of PRES-SCI in renal disease, including the present case (IgA nephropathy (n=3), membranoproliferative glomerulonephritis (MPGN) (n=2), lupus nephritis [n=1], acute glomerulonephritis after streptococcal infection (n=1), and chronic kidney disease (CKD) of unknown details (n=3)) (Table 2) [7,14-19].

**Table 2:** Summary of 10 cases of PRES-SCI in renal disease, including our case. IgA: Immunoglobulin A; BP: blood pressure; CKD: chronic kidney disease; MPGN: membranoproliferative glomerulonephritis; IVMP: intravenous methylprednisolone pulse; MMF: mycophenolate mofetil.

Case	Authors	Age	Sex	Diagnosis	Clinical symptoms	Immunosuppressant	Spinal lesion	From onset to treatment	Therapy	Residual symptoms
1	Choh et al. [14]	17	M	IgA nephropathy	Headache, vomiting, visual disturbance	-	Cervical	A few days	BP reduction	None
2	de Havenon et al. [7]	50	M	CKD, untreated hypertension	Headache, vomiting, lower limb muscle weakness, disturbance of consciousness, visual disturbance	-	Cervical Thoracic	1 Week	BP reduction	Mild lower limb muscle weakness
3	de Havenon et al. [7]	25	M	MPGN, untreated hypertension	Headache, visual disturbance	-	Cervical	2 Weeks	BP reduction, IVMP	None
4	Chen et al. [15]	16	F	MPGN	Headache, vomiting, visual disturbance, lower limb muscle weakness, seizure, urinary incontinence, disturbance of consciousness	-	Cervical Thoracic	1 Week	BP reduction	None
5	Chen et al. [15]	8	M	Poststreptococcal glomerulonephritis	Headache, vomiting, visual disturbance, lower limb muscle weakness, seizure, urinary incontinence	-	Cervical Thoracic	5 Days	BP reduction	None
6	Liu et al. [16]	20	M	CKD, uncontrolled hypertension	Headache, visual disturbance, limb muscle weakness	-	Cervical Thoracic	1 Week	BP reduction	None
7	Prabhahar et al. [17]	28	M	IgA nephropathy	Headache, vomiting, visual disturbance, seizure	-	Cervical	1 Week	BP reduction	None
8	Ayvacioglu Cagan et al. [18]	23	M	CKD, untreated hypertension	Headache, vomiting, visual disturbance	-	Cervical	10 Days	BP reduction, hemodialysis	None
9	Ghaderi Yazdi [19]	18	F	Lupus nephritis	Headache, nausea, visual disturbance, seizure, disturbance of consciousness	Cyclophosphamide	Cervical	-	BP reduction	None
10	Present case	43	M	IgA nephropathy, living donor kidney transplantation, uncontrolled hypertension	Headache, vomiting, visual disturbance	Tacrolimus, MMF	Entire	18 Days	BP reduction, hemodialysis, IVMP	None

This case was the only one with post-renal transplantation. Muscle weakness was observed in four patients [7,15,16]. Immunosuppressive drugs were used in two patients, including the patient in this case [19]. Cervical cord involvement was common, and entire spinal cord involvement was observed only in this case. The time from disease onset to treatment was the longest in this case. All patients were treated with antihypertensive therapy. Hemodialysis was administered in two cases, including the present case. While withdrawal from hemodialysis was impossible in Case 8 [18], our patient was weaned off hemodialysis. The prognosis was good in most cases, but one patient had residual mild muscle weakness in the lower limbs [7]. This may be because the patient was of relatively advanced age and had muscle weakness due to a spinal cord lesion from the onset. In this case, worsening renal function due to rejection after renal transplantation led to the development of malignant hypertension. Both malignant hypertension and immunosuppressive drugs may have contributed to the development of PRES-SCI. Although our patient had more risk factors for PRES and a longer time to intervention than previously reported cases, the relatively young age at onset and the absence of limb weakness at onset may have contributed to a good outcome.

## Conclusions

This is the first case of PRES involving the entire spinal cord in a patient with kidney disease. Malignant hypertension, post-transplant rejection, and the use of immunosuppressive medications may contribute to the onset of PRES-SCI. Although PRES is rarely accompanied by spinal cord lesions in patients with kidney disease, PRES-SCI can occur in these patients with kidney disease, and prompt diagnosis and treatment may lead to a good outcome.
